# Diet of Black-backed Jackal (*Canis mesomelas*, Schreber, 1775), impacts on livelihood and perceptions of farmers in Konasa Pulasa community conserved forest, omo valley of Ethiopia

**DOI:** 10.1186/s40850-023-00186-5

**Published:** 2023-11-09

**Authors:** Mesfin Matusal, Aberham Megaze

**Affiliations:** Department of Biology, Wolaita Sodo University, P.O. Box 138, Wolaita Sodo, Ethiopia

**Keywords:** Conflict, Community-based forest, Diet, Livestock, Depredations, Mitigation

## Abstract

**Background:**

Livestock depredation by the black-backed jackal (*Canis mesomelas*) occurs widely across Africa. The study on human-jackal conflict is important for conservation efforts in Ethiopia. The aim of this study was to investigate the diet of black-backed jackals, to understand their predation effects on domestic livestock and perceptions of farmers’ in the Konasa-Pulasa Community Conserved Forest, Omo Valley of Ethiopia. The study was conducted using scat analysis, questionnaire survey and Focus Group Discussion methods. Livestock depredation and the economic impact of farmers were assessed among 290 randomly selected households. A total of 90 scat samples were collected and analyzed during the dry and wet seasons to identify the diet of jackals.

**Results:**

A total of 624 domestic animals have been lost in the last 5 years (2016–2020). The estimated economic cost of domestic animals lost due to predation by jackals was US $18,180.0 in the last five years, and US $12.5 per year per household. The major diet composition of jackals was of domestic animal origin (45.5%), followed by wild animals (30.8%) in both the dry and wet seasons. However, more prey diversity was recorded during the wet season. The respondents revealed that the causes of black-backed jackal conflict in the study area were higher due to increasing jackal population size (40%). The major traditional mitigation method was guarding (42%). Most of the respondents (48.2%) had negative perceptions towards the conservation of black-backed jackals.

**Conclusion:**

Livestock depredations by black-backed jackals were the major issue of conflict in the study area. Scat analysis showed that large percentage of domestic animal species remain in the scat of jackals. Livestock losses caused by jackals represent an economic concern for livestock owners in the area. Local people close to the forest boundary were highly vulnerable to domestic animal loss due to predation by jackals. Therefore, improved livestock husbandry methods will be implemented by the local people for effective jackal conservation in Konasa-Pulasa Community Conserved Forest. Understanding the ecological and social dimensions of conflict situations in the area may have important ecological and management implications for the country.

## Introduction

Human − carnivores conflict is one of the most widespread and intractable issues facing conservation biologists today [1–3]. Conflicts become more intense when livestock holdings and agriculture are important parts of rural livelihoods [4, 5]. It is not only occurring in Africa, but it is also a global problem in many countries where human and wildlife requirements overlap [5, 6]. All continents and countries, whether developed or not, are affected by the human-carnivore conflict. Developing countries are more vulnerable than developed nations where livestock rearing and crop cultivation are important parts of rural people’s livelihoods, including Ethiopia [7]. Globally, livestock depredation by apex and meso-predators is a fundamental challenge in many developed and developing countries [8].Domestic animal predation is a major carnivore conservation challenge in the KPCCF, causing significant economic losses and frequently provoking retaliatory killing [9].

The spotted hyena (*Crocuta crocuta*), black-backed jackal (*Canis mesomenalis*), and caracal (*Caracal caracal*) are commonly reported sympatric apex and mesopredators, and they detect live prey and leftovers by sight and hearing through scavenging and hunting [10, 11].

These predators can contribute to food insecurity, income problems for farmers living near protected areas, and reduce tolerance for species. Around the KPCCF, domestic animal depredation is a major carnivore conservation challenge [9]. Perhaps the two most important factors contributing to domestic animal predation in the area are the high human density and the decrease in wild prey. In contrast, potential dangers posed by wild animal species negatively influence the perception of local people towards wildlife. The perception of local people towards wildlife conservation is likely to be influenced by personal beliefs, but there are many other factors such as cultural norms, societal experiences, education, age, different experienced costs and benefits and other related factors [11–13].

The black-backed jackal (hereafter named ‘jackal’) is a widely distributed medium-sized carnivore that exists in most protected areas of Africa and tolerates a wide range of habitats, from arid coastal desert to montane grasslands, open savanna, arid savanna, woodland savanna mosaics, farmlands, and villages [14–17]. For many years, little was known about medium-sized carnivores such as jackals in various parts of the world, including Ethiopia. They have not attracted the attention of conservationists due to their minor impacts on local people living close to the forest in terms of livestock depredation. They were the least concerned about the International Union for the Conservation of Nature (IUCN) Global Red List of Threatened Species because these animals remain widespread in Africa [17, 18].

Jackals are generalist feeders, consuming mostly small and medium-sized mammals, birds, carrion, insects, and fruit. However, in some areas, larger wild ungulates and domestic ungulate species are consumed. Jackals have the ability to modify their diet in response to variations in resource availability [3, 16, 19]. [20] mentioned that jackals follow an optimal foraging pattern that allows them to opportunistically access spatially and temporally variable resources. Jackals are infamous for their adaptable behaviour as a response to human activity [21]. Jackals have a long history of conflict with farmers in South Africa. Domestic and wild ungulates were important sources of food for jackals in South Africa and Botswana [22, 23]. The dietary analysis in [14, 15] revealed that jackals feed on a wide variety of food items such as domestic and wild mammals, plant materials, anthropogenic items, insects, and birds across different habitats.

The diets of the apex and meso carnivores reflect both the availability of potential prey and a suite of morphological, behavioral, and physiological adaptations that allow the individual to locate, capture, ingest, and digest a diverse range of prey taxa [2, 3]. However, the degree to which the relative frequencies of identifiable remains represent the actual proportion of prey types eaten is unknown [24, 25]. Identification of prey hair from medium-sized wild carnivore provides a reasonable basis for diet reconstruction. Different mammals have hair of different lengths, thicknesses, and macrostructures, affecting the hair’s ability to be broken down by digestive enzymes [15, 26].

Scat analysis is a widely used technique to assess carnivore diets, because the approach is inexpensive, non-invasive, relatively quick to apply, and large samples can be collected [27]. It is a useful tool to construct a basic description of a carnivore’s diet, particularly when other methods are difficult to conduct. Because scat analysis is an indirect method, results could be biased by various factors, such as partial or total digestion of remains, misidentification of food remains, and under representation of smaller prey items [28]. The method is compatible with the endangered status of many carnivores in different countries [17, 29].

A wide range of management tools have been developed worldwide to address human-carnivore conflicts, but most are strongly site and species/genera specific and are not widely or easily accessible [4, 7, 30]. Most communities use their indigenous knowledge to mitigate the effects of human-carnivore conflict, particularly jackals. This provides insight into how different communities survive where wildlife, people, and livestock interact and compete for the same natural resources [31, 32]. The scenario is the same in southern Ethiopia, with diverse biological resources [33].

In southern Ethiopia, very little investigations were conducted to assess conflict between humans and jackals and focused on the role of jackals as predators of livestock [15]. At present, there is no data on human- jackal conflict in the KPCCF. Effective conservation measures cannot be achieved successfully in the absence of clear information about the ecology of the species. The farmers in the study area complain of livestock losses due to the impact of jackals in and around the KPCCF. Understanding the levels of jackals perceived and actual livestock predation is important to designing appropriate measures to reduce livestock loss. Conducting scientific research on human-jackal conflict in the area would provide a valuable solution to minimize the existing problem and create a better coexistence between humans and jackals. Therefore, this study focused on identifying the preferred prey items from the diet of jackals, both domestic and wild ungulates, the economic loss of livestock caused by jackals; mitigation methods used by local people; and the perceptions of farmers living in and around KPCCF in the Omo Valley of Ethiopia. Knowledge of the food habits of the jackals in the study area will provide basic ecological information and elucidate the reasons for jackal predation on domestic stock and could provide insight into decreasing conflict and ensuring the persistence of the animals in the area.

## Materials and methods

### Study area

**Fig. 1:**
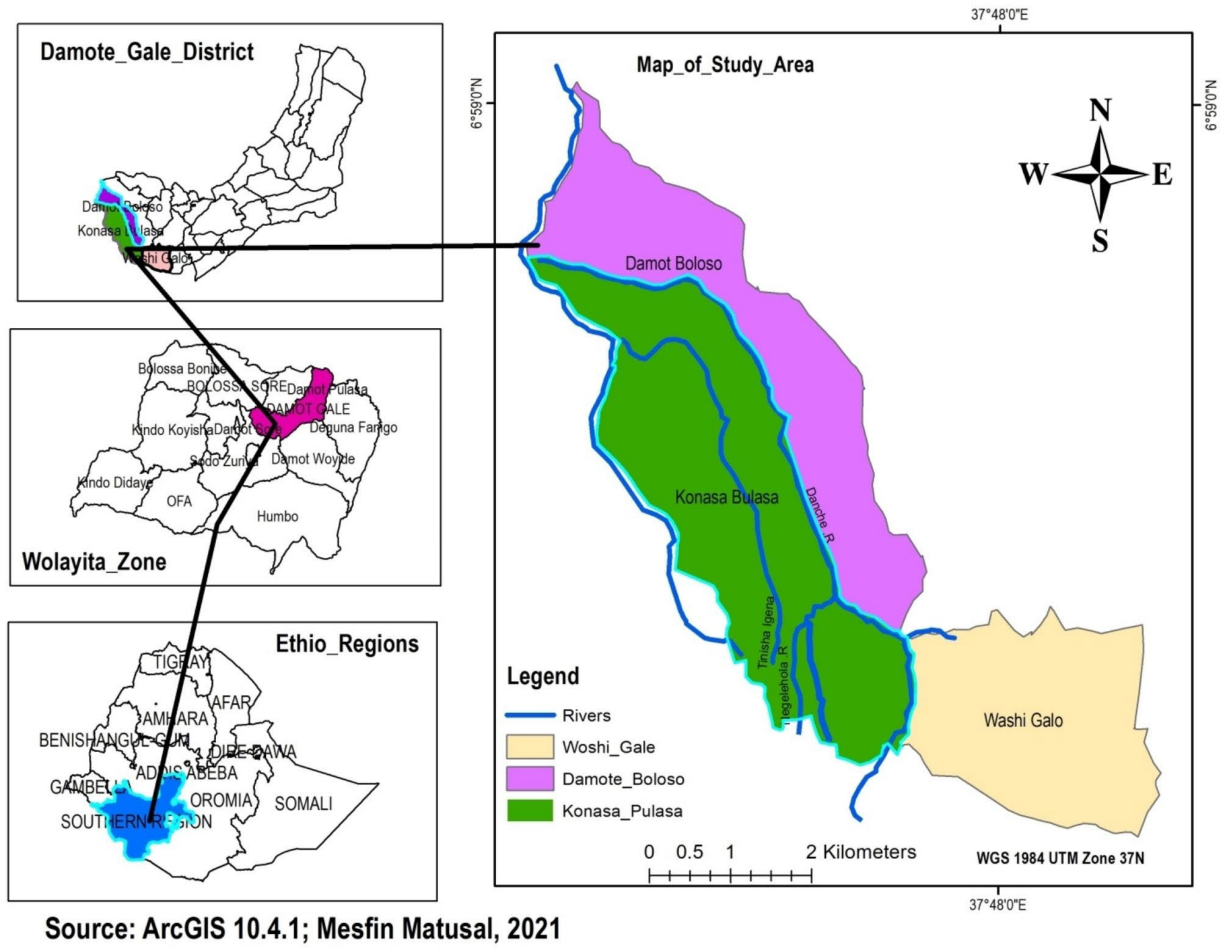
Map of the study area

The study was conducted in the Konasa-Pulasa Community Conserved Forest (hereinafter, “KPCCF”). The area is in the north longitudes between 37^0^ 42’ 30” to 37^0^ 50’ 30” and east latitudes 6^0^ 50’ 30” to 6^0^ 58’ 00” with an altitudinal range of 1500 to 2950 m.a.s.l. (Fig. 1). The study area covers, 187.57 km^2^. It is located in Damot Gale District, Wolaita Zone, Omo Valley of Ethiopia, 364 km south of Addis Ababa, Ethiopia’s capital city.

The local people of KPCCF depend on mixed farming (crop cultivation and livestock rearing such as poultry, sheep, cattle, horses, mules, and donkeys). The livestock are kept inside the house at night. They are kept outside the home on the grazing land or at KPCCF throughout the daytime, between 8:00 a.m. and 18:00 p.m. The mean annual rainfall ranges between 1450 and 1800 mm. The area receives its maximum rainfall between June and September, while the minimum rainfall is between March and April. The annual minimum and maximum temperatures in the area are 16 and 24 °C, respectively [34]. The dominant soil color of the study area was pale brown, reddish, and dark in color, clay, and loam in texture [35]. The Yichia, Etana, Kaleta, and Beshir rivers and their tributaries drain the area. These rivers then flow to Abaya Lake and the Omo River; they are the largest Rift Valley lake and river in southern Ethiopia.

The study area is also characterized by rugged topography, diversified agro-ecology, fauna, and flora. The vegetation of KPCCF are diverse and dominated by various plant species such as *Juniperious procera, Acacia bervispica, Croton macrostachyus, Euphorbia tirucelli, Syzygium guineense, Hagenea abyssinica*, *Cordia Africana, Croton macrostachyus, Albizia gumifera, Allophylus abyssinicas, Erica arborea, Olea europaea, Cupressus lusitanica* and *Bambusa* [35]. The area has rich faunal biodiversity and consists of large and medium sized mammal species such as Anubis baboons (*Papio anubis*), grivet monkey (*Cercopithecus aethiops*), bushbuck (*Traglapus scriptus*), common duiker (*Sylvicapra grimmia*), hare (*Lepus timidus)*, guenther dikdik (*Madoqua guentheri*), porcupine (*Hysteric cristata*), bush hyrax *(Heterohyrax brucei)*, aardvark (*Orycteropus afer*), and predators include black backed jackals (*Canis mesomelas*), genets (*Genetta* genetta), white tailed mongoose (*Galerella flavescens*), leopards (*Panthera pardus*), serval cat (*Leptailurus serval*), African civet (*Civetticis civetta*), spotted hyena (*Crocuta crocuta*) and different species of birds [35]. The socio-economic condition of the people in the study area is mainly agro-pastoralist. Subsistence farming is the main source of income for the local community. The main crops are cereals such as maize (*Zea mays*), sorghum (*Sorghum bicolor)*, teff (*Eragrostis tef)*, pea (*Pisum sativum*), beans (*Faba vulgaris)*, wheat (*Triticum aestivum*), barley (*Hordeum vulgare*), cash crops like coffee (*Coffea arabica L.*), Taro (*Colocasia esculenta*), banana (*Musa* sp.), false banana (*Ensete ventricosum*) and sweet potato (*Ipomoea batatas*) and fruits like banana (*Musa paradisca L*.), mango (*Mangifera indica*) and avocado (*Persea americana*).

### Study design

The present study used a questionnaire survey method among the households and scat analysis techniques to assess livestock depredation by jackals during 2016–2020. In the questionnaire survey, semi-structured questions with both closed and open-ended answers were used. Interviewees were also able to contribute their own personal experiences and elaborate on topics that may have been too constrained in a highly structured questionnaire format [36]. The three Peasant Associations (Konasa Pulasa, Woshi Gale and Damot Boloso) were purposefully selected among seven Peasant Association around KPCCF (Fig. 1). Five villages were purposefully selected from the three Peasant Association (Wonchiro, Mehal, Damota, Sorto, and Sutancho), these villages are nearer to the forest and face the problem earlier than the others. The villages were divided into three groups based on their distance from the KPCCF edge: close (< 1 km), medium (1.5–2 km), and far (> 2.5 km).

The questionnaire was pretested on 45 randomly selected individuals from all five villages of varying age, sex, and background among the local communities, not included in the main sample group. This helped to modify the questionnaire accordingly [4]. The pretested questionnaires were used in examining the practicability, reliability, and suitability of the method. The sample size of the study population was selected among 1043 households from five villages. Systematic random sampling techniques were used to select the households based on the simplified formula developed by [37].

$$\text{n}=\frac{N}{1+N\left({e}\right)^{2}}$$

Where: n = sample size, N = is the total population size, e = is the level of precision,

$$\text{n}=\frac{1043}{1+1043\left(0.05\right)^{2}}=290$$

Among 290 sample size, 83.7% (n = 243) were male and 16.2% (n = 47) were female respondents.

The data was collected from December 2019 to May 2020, following the procedures adopted in [38–40]. The questionnaires were administered to the households during the wet and dry seasons of 2019 to 2020. The questionnaire was prepared in English and translated into Wolaitato to easily communicate and reduce misunderstandings during the interviews. The questionnaires were administered randomly to members of the households based on a first-come, first-serve basis. The household representatives included both females and males of various age groups. The age category of participants ranged from 18 to 80 years old. An interval of 35–50 min was required for the interview [16]. All the household questionnaires focus on three main areas of interest: These include: (i) demographic and socioeconomic information of respondents (ii) identification of prey species, losses of livestock to jackal killed, when and where attacks on livestock occurred and livestock lost to other causes of death and their economic loss from 2016 to 2020 were covered, (iii) mitigation methods to reduce livestock losses and perceptions of local people towards the conserved forest and jackals, following the approach used by [12]. The perception of respondents for jackal was categorized as: a major problem (negative perception), a minor problem (negative perception), no problem (positive perception) and no response (neutral). Each perception statement is stated according to their strength of agreement by a five-point Likert scale [41]. Two Focus Group Discussion sessions were conducted after the questionnaire to support the questionnaire data. The group size in each discussion site (village) varied from 7 to 15 people. A checklist of questions was developed to guide the Focus Group Discussions to obtain first-hand information from participants [39]. The participants were invited to discuss issues according to their convenience. District agricultural offices, village leaders, local elders, model farmers, peasant females association, and students were participated to discuss their experiences concerning causes of human-jackal conflict and their mitigation, the management style of the species, and perceptions of informants towards community conserved forest [10]. The discussions were done with the aid of the two trained research assistants. Direct field observation was also important in identifying the particular problems in the study area. In order to estimate the extent of livestock damage by jackals, the teeth marks left on body parts of livestock, scats, foot-marks, and dens were used. During the survey, all the damaged domestic animals were recorded. In addition, GPS location and digital photographs were taken. Finally, the mean loss of domestic animals was estimated, compared with the current average market price of the area and supports the reported depredations obtained from interviews [40, 42]. Laboratory examination of jackal scat from the study area supported the data gathered through questionnaires and focus group discussions.

### Method of scat collection and laboratory procedure

Jackal scats were collected during the dry and wet seasons from December 2019 to May 2020 in order to assess jackal food habits and better understand the conflict situation. Scats were collected according to opportunistic sampling along road transects. Scat was distinguished from other sympatric carnivores by its size, shape (long with pointed ends), color, tracks, and contents [43], as well as its proclivity to deposit scats on conspicuous objects such as rocks and tufts of grass along roads and paths [44]. During the collection of in the field, scat sample were put in zip-lock plastic bags and labeled with date, codes of each scat, GPS locality, estimated age of scat, and villages were recorded. A totally of 90 scats of the jackals were collected from the KPCCF in the dry (n = 43) and wet (n = 47) seasons during the sampling period.

Following the collection of samples, all scats were soaked in a 5% formalin solution for > 24 h to kill potential parasites. Samples were oven dried at 50 °C for 48 h. Food types consumed by jackals were determined by identifying undigested remains in their scats. Each dried scat was then spread over a 20 × 20 cm grid in order to estimate the proportion of each prey category. A magnifying glass was also used to identify food remains. The presence of any materials such as, hair, bone, feathers, plant materials, etc. was identified and recorded [45]. The scale morphology of mammalian hairs is often of interest to zoologists, especially for use in species identification in the scat sample. Hence, hairs were washed and cleaned in alcohol for 1–3 h, soaked in ethanol solution for a minimum of 5 min, and dried on filter paper. The cuticle slides were made by soaking 1.7 g of gelatin (one sheet of Oetker white) in 10–40 ml of demi-water for 5 min. The solution was then placed on a hot plate (65 °C) until the gelatin to dissolve. The dissolved gelatin cooled after 5 min. A thin layer of gelatin solution was then spread on a microscope slide and left to dry for 5 min. The hairs were placed on the gelatin layer and left until the gelatin was completely dry (minimum 60 min). When the gelatin was dry, the hairs were removed with forceps. The cuticle imprints were examined and photographed by compound microscope. Hairs were identified according to cuticle scales and medulla patterns (texture, color, and width, banding patterns) [46].

For mammal species identification, ten hairs were removed at random for microscopic hair scale imprint identification [47, 48]. These hairs were then mounted on a slide and removed when dry to derive the hair scale imprint. The hair was kept for macroscopic examination in the Zoology Laboratory of Wolaita Sodo University. The microscopic slides with imprints are examined at 4x to locate the hair and then further scrutinized at 10x and 40x objectives. The imprint was inspected from the proximal to the distal end. The prey species were identified through hair analysis techniques. Hairs recovered from scats were identified using the published manual [46, 49]. Comparisons were made with a photographic reference of hairs from east African species and the reference manual of [46].

The percentage occurrence of six categories of food was recorded from the scat of jackals: domestic mammals, wild mammals, insects, plant materials, anthropogenic items, and unidentified items. In some cases, an item could be recorded as “unknown items.“ These were all grouped under ‘unknown’ category. Teeth, jaw fragments, skull and other bone fragments, hairs, and other small vertebrate remains in scats were used to identify the remains. Using the remnants’ of claws, beaks and feathers, avian remains were identified [50]. Anthropogenic food items were also identified from the presence of oddities (bone chips, plastic, paper and string), and unidentified items [51]. We assessed jackal diet composition by using the frequency of occurrence, percentage frequency of occurrence, and relative percentage frequency of occurrence of prey items in the total number of scats collected [23]. Although this method is well suited to identify rare prey groups, it does not fare well when ecological questions regarding the impact on prey species are posed [48].

### Data analysis

Data analysis consisted of both descriptive and inferential statistics. ANOVA was used to test the statistical difference in means of variables of the total number of livestock type lost to jackals in villages and economic costs associated with the losses of livestock, in addition, the nonparametric χ^2^ tests was used to test the observed frequency of predation on different types of livestock between villages, perception of the respondents on problem animals and seasonality in depredation. A correlation was also done to determine the relationship between livestock damage and distance from the forest. The difference in the occurrence of food items of jackals was tested with Pearson chi-square test. The effects of socioeconomic variables on participants’ perceptions were investigated as continuous variables such as Gender, age, education status, family size, farm land size, farming practice were converted into categorical variables following [52] to compare the effect within a group. Perceived jackal depredation was used as dependent variables, while six socioeconomic variables were used as independent variables. Descriptive statistics were used in the form of percentages and frequencies to analyze the respondents’ demographic and socioeconomic profiles. It was also used to compare the frequency of occurrence and percentage of prey species analysed from the jackals diet. The predation control methods practiced by the local people were analyzed using descriptive statistics. The information was collected from Focus Group Discussion, summarized by text analysis, and presented narratively and used to supplement the perceptions of the household respondents. All analyses were conducted using the Statistical Package for Social Science (SPSS) version 23.0 for Windows. All statistical tests were two-tailed, with a significance level of *p* ≤ 0.05. Average prices of different livestock types (cattle US$72.17, sheep US$22.15, horse US$42.51, mule US$ 24.00 and donkeys US$15.90 and poultry US$ 9.55) were computed using different market prices that livestock were sold at as given by respondents and used to calculate the cost of livestock lost to black-backed jackals. Values of economic losses are discussed in US$, using the exchange rate of 1 US$ = 40 ETB at that time.

## Results

### Socio-demographic profile of respondents and their perception towards the conservation of black backed jackals

Of the 290 households respondents, 83.7% (n = 243) were males and 16.2% (n = 47) were females, and gender varied significantly among respondents (χ^2^ = 120.4, df = 1, P < 0.05), but there was no significant difference in the perceptions of male and female respondents towards the conservation of jackals. Among sample respondents, 18% were young age 18–30 years old, 36% of them were between the ages of 31–45 years old, 25% of them were between the age of 46–61 years old, and 21% of respondents were 61–80 years old. Young (18–29 years old) 30 (58.8%) and middle age (31–45 years old) 64 (60.4%) respondents showed more positive perception towards the conservation of jackals in the study area. (χ^2^ = 77.3, df = 4, P < 0.05).

Among the respondents (33%) were illiterate, and 39% were completed primary education. There was a significant difference in educational status among the respondents (χ²=151, df = 3, p < 0.05). Moreover, the larger proportion of the educated respondents (54.1%) had positive perception towards the conservation of jackals. Relatively, better-educated groups expressed positive perception than uneducated respondents.

Most (75.9%) respondents had 5–9 family members. Respondents with large family size (71.8%) had negative perception towards the conservation of jackals than those with small family size (17.1%). Mixed farming (crop cultivation and livestock rearing) were the main means of livelihood in the study area. Agro-pastrolism (mixture of rearing livestock and crop farming) was practiced by 67.2% of the respondents while 17.9% practiced only crop farming. The respondent (52.3%) who depends on mixed farming had a negative perception towards the conservation of jackals.

Among the respondents, 52.8% have 0.5-1 hectare farmland, 16.9% were 2.6-4 hectare, and 3.8% was 4.1 and above hectare. Relatively, large hectares of farmland respondents expressed positive perception (54.5%) towards the conservation of jackals than small sized farmland respondents (36.6%)(Table 1).

**Table 1:** Socio-demographic profile of respondents and their perception towards the conservation of jackals in Konasa-Pulasa Community Conserved Forest

Socio-demographic profile			Perceptions about conservation of black-backed jackals (%)					
Variables	n = 290	%	Positive	Neutral		Negative		
Gender								
Male	243	84	136 (55.9)	10 (4.1)		98 (40.3)		
Female	47	16	20 (42.6)	12 (25.5)		15 (31.9)		
Average			49.3%	14.8%		36.1%		
Age categories								
18–30 years old	51	18	30 (58.8)	4 (7.8)		17 (33.3)		
31–45 years old	106	36	64 (60.4)	7 (6.6)		35 (33.0)		
46–60 years old	73	25	36 (49.3)	13 (17.8)		24 (32.9)		
61–80 years old	60	21	17 (28.3)	9 (15.0)		34 (56.7)		
Average			49.2%	11.8%		38.9%		
Educational status								
Illiterate	95	33	30 (31.6)	21 (22.1)		44 (46.3)		
1^0^ education ( 1–8)	113	39	40 (35.4)	20 (17.7)		53 (46.9)		
2^0^ education (9–12)	59	20	42 (71.2)	5 (8.5)		12 (20.3)		
Above 12 grade	23	8	18 (78.3)	2 (8.7)		3 (13.0)		
Average			54.1%	14.5%		31.6%		
Family size								
1–4	70	24.2	50 (71.4)	8 (11.4)		12 (17.1)		
5–8	117	40.3	48 (41.0)	15 (12.8)		54 (46.2)		
9 and above	103	35.5	23 (22.3)	6 (5.8)		74 (71.8)		
Average			44.9%	10.0%		45.0%		
Farming practice								
Mixed farming	195	67	77 (39.5)	16 (8.2)		102 (52.3)		
Crop cultivation	52	18	25 (48.1)	4 (7.7)		23 (44.2)		
Livestock rearing	41	14	5 (12.2)	3 (7.3)		33 (80.5)		
Other activity	2	1	2 (100)	0 (0.0)		0 (0.0)		
Average			50.0%	5.8%		44.3%		
Size of farm land								
0.5-1 hectare	153	52.7	56 (36.6)	14 (9.1)		83 (54.2)		
1.1–2.5 hectare	77	26.5	32 (41.6)	4 (5.2)		41 (53.2)		
2.6-4 hectare	49	17.0	20 (40.8)	5 (10.2)		24 (48.9)		
4.1-above	11	3.8	6 (54.5)	1 (9.1)		4 (36.4)		
Average			43.4%	8.4%		48.2%		

### Depredation of domestic animals and economic loss by black-backed jackal

Depredation of domestic animals by jackal’s species occurred in all the study villages, but high depredations were occurred in the villages close to community conserved forests. A total of 624 losses of domestic animals to jackal’s predation were recorded in the last five years. The highest number of kills was reported for sheep representing 31.7% (n = 198) of the total domestic animals killed by jackals, followed by the poultry (30.8%, n = 192) and cattle (19.2%=120) while the donkey was the least (5.3%, n = 33). The livestock loss caused by jackals was also significantly differed across the study villages (F42 = 2.75, df = 4, p < 0.05). The degree of conflict was reported to be more intensive within 0.5 to 2 km range from the forest than within the distance villages (r = 0.71, P < 0.05). With increasing distance from the forest boundary, predation on livestock decreased. The total economic cost attributed to livestock losses by jackals predation amounted to US$18,180.00 with cattle accounting for 47.63% (US$8,660.00), sheep 24.12% (US$4,385.00), and donkeys 2.89% (US$525.00). The estimated annual costs due to the loss of domestic animals were US$ 3,636 per year, which was US$ 12.5 per household per year. There was a significant difference between domestic animal species consumed by black-blacked jackal (χ²=27.2, df = 5, p < 0.05)(Table 2).

**Table 2:** Domestic animal species depredated by jackal and their economic valuation in villages of KPCCF, during 2016–2020 in the Omo Valley of Ethiopia, on the basis of interviews (n = 290)

Species predated	No of stock	Villages					Total kill	Total loss in US$
		Wonchiro (< 1 km) (n (%))	Mehal (1.5-2 km) (n(%))	Damota (1.5-2 km) (n(%))	Sorto (> 2.5 km). (n(%))	Sutancho (> 2.5 km). (n(%))		
Sheep	976	72(33.33)	54(34)	31(27)	25(31)	16(30)	198	4,385.00
Poultry	1554	73(33.7)	45(28)	34(29)	23(29)	17(32)	192	1,834.00
Cattle	728	37(17)	28(18)	24(21)	18(23)	13(25)	120	8,660.00
Mule	233	13(6)	9(6)	7(6)	5(6)	2(4)	36	863.00
Horse	275	11(5)	16(10)	11(9)	4(5)	3(6)	45	1,913.00
Donkey	357	10(4.6)	7(4)	9(8)	5(5)	2(5)	33	525.00
Total	4,123	216	159	116	80	53	624	18,180.00

Foot note: 1 US$=40 ETB, n = number of killed domestic animals

### Seasonality of domestic animal species depredation

**Fig. 2:**
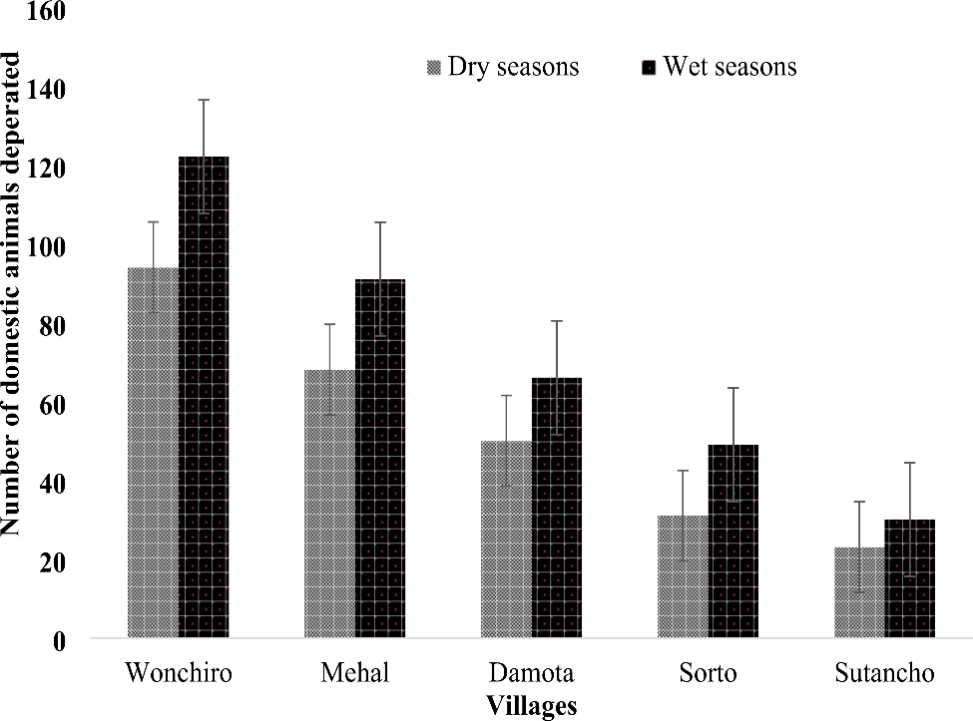
Seasonality of domestic animal depredation in Konasa^_^Pulasa Community Conserved Forest, Omo Valley of Ethiopia from 2016–2020

Among 624 domestic animals killed, 44.1% (275) were killed in the dry season (December–February 2019), and 55.9% (349) were killed in the wet season (March–May 2020). There was a significant difference between seasonality of depredation (χ²=16.5, df = 1, p < 0.05). Domestic animal depredation has occurred in villages close to the forest, such as Wonchiro (34.6%, 216), Mehal (25.5%, 159), and Damota (18.6%, 116) (Fig. 2).

### The causes of human–black backed jackal conflict

Most of the participants (117, 40%) responded that the causes of human– jackal conflict in the study area were due to increasing of the jackals population size, followed by resource computations (103, 36%) between human and jackals. There was a significant difference between participant responses (χ²=65.7, df = 2, p < 0.05) (Table 3). Focus Group Discussants suggested that the number of jackals should be reduced from the by removing from the area or by killing them, compensation for inhabitants of the forest and awareness creation to know how to reduce jackal predation.

**Table 3:** The causes of human black-backed jackal conflict around the Konasa^_^Pulasa Community Conserved Forest in Omo Valley of Ethiopia, on the basis of interviews (n = 290)

Causes of human black-backed jackal conflict	Respondents	
	Frequency	Percent (%)
Increase black-backed jackal population in the area	117	40
Resource competitions between human and jackal	103	36
Habitat loss or fragmentation	70	24
Total	290	100

### Diet composition of black-backed jackal

A total of 90 scats samples of jackals were collected and identified in the dry and wet seasons (Table 4). Out of 90 collected scats, 48% (n = 43) were collected in the dry season, and 52% (n = 47) scats were collected during the wet season. A total of 149 and 205 sample slides were analyzed in dry and wet seasons, respectively. Six different types of prey items were identified from diet analysis of jackals. Large portion of the diet composition of jackals were domestic animals (45.5%, 161), followed by wild animals (the principal prey items) (30.8%, 109), insects (10%, 35), plant materials (7.6%, 27) (seeds of wheat and maize, stems of unidentified plant species), fruits and vegetables), (5%, 19) were anthropogenic food items (plastic material, clothes, soil) and (0.8%, 3) were unidentified prey species in both the dry and wet seasons. The frequency of occurrence of prey items in the diet was significantly differed (χ^2^ = 134, df = 5, P < 0.05). Among identified prey species in the diet of jackals, rodents, poultry, and sheep frequency of occurrence constitute 20.0%, 18.6%, and 16.4%, respectively. The Ackerman index shows rodents, sheep and poultry were the most preyed items. Similarly, the Ivelevis index shows that rodents (+ 0.3), poultry (+ 0.06), and sheep (+ 0.25) were the preferred items for the jackals.

**Table 4:** The seasonal relative frequency of occurrence of prey species remains (%) in 90 jackal scat sample collected in KPCCF, Omo Valley of Ethiopia (December 2019 to May 2020). n = number of scats; N = number of specific prey items

Food items of black-backed jackals	Seasons		
	Dry (n = 43)	Wet (n = 47)	Total (n = 90)
Domestic mammals, 45.5% (N = 161)	N (%)	N (%)	N (%)
Sheep	27 (18.1)	31(15.1)	58(16.4)
Poultry	30(20.1)	36(17.6)	66(18.6)
Cattle	10(6.7)	22(10.7)	32(9.0)
Donkey	1(0.7)	2(1.0)	3(0.8)
Horse	1(0.7)	1(0.5)	2(0.6)
Wild mammals, 30.8% (N = 109)			
Rodents	40(26.8)	31(15.1)	71(20.0)
Hare	7(4.7)	14(6.8)	21(5.9)
Duiker	3(2.0)	11(5.4)	14(4.0)
Bush buck	0(0.0)	2(1.0)	1(0.3)
Birds	0(0.0)	1(0.5)	1(0.3)
Insects, 10%(N = 35)			
Aphids	4(2.7)	10(4.9)	14(4.0)
Grasshopper	5(3.4)	7(3.4)	12(3.4)
Beetles	3(2.0)	4(2.0)	7(2.0)
Wasps	2(1.3)	0(0.0)	2(0.6)
Plants materials, 7.6% (N = 27)			
Seeds	5(3.4)	11(5.4)	16(4.5)
Vegetables	3(2.0)	5(2.4)	8(2.3)
Fruits	2(1.3)	1(0.5)	3(0.8)
Anthropogenic items,5% (N = 19)			
Soil	3(2.0)	7(3.4)	10(2.8)
Plastic	3(2.0)	5(2.4)	8(2.3)
Clothes	1(0.7)	0(0.0)	1(0.3)
Unidentified preys, 0.8%(N = 3)	0(0.0)	3(1.5)	3(0.8)
Total number of prey items, (∑N)	149(42.1)	205(57.9)	354(100)

### Local predation-mitigation techniques

**Fig. 3:**
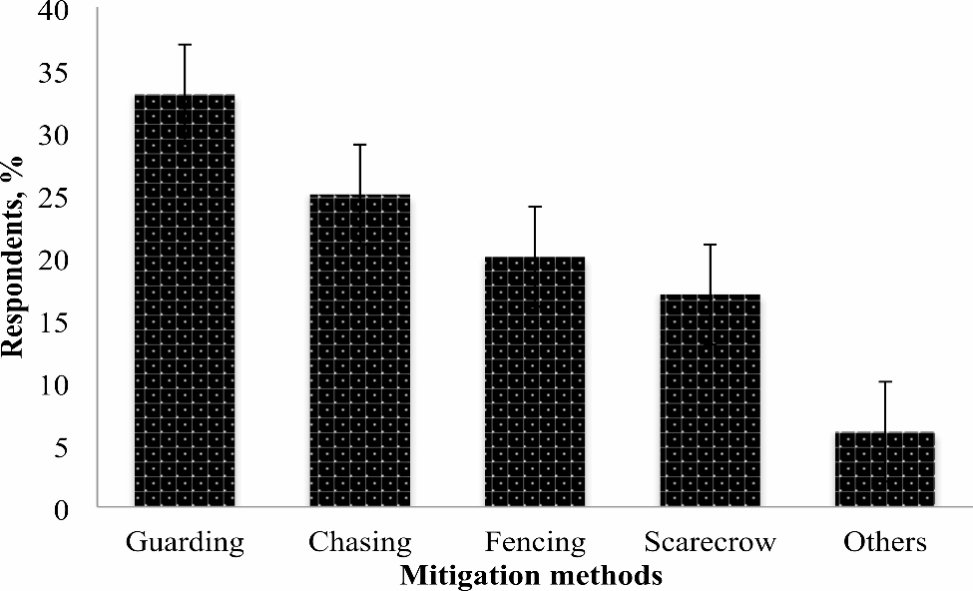
Mitigation methods to deter livestock depredation in and around the Konasa^_^Pulasa Community Conserved Forest

Farmers utilize indigenous methods to mitigate their domestic animals. Most of the respondents, 33% (95) in the study villages, responded that guarding was the most effective strategy in order to protect domestic animals, followed by chasing 25%(73), fencing 20%(57), scarecrows 17%(49), and 6%(16) were used other traditional techniques (Fig. 3). There was a significant difference in mitigation methods used by a local farmer (χ^2^ = 93.724, df = 4, p < 0.05).

## Discussions

The combination of socio-economic questionnaires and scat analysis results showed that human-jackal conflict is one of the major issues facing the farmers living around KPCCF. Jackals in the KPCCF consume a high proportion of domestic ungulates compared to other prey items. The abundance of domestic ungulates in the diet, accounting for a large percentage of the food items consumed, resulted in stable diet that did not differ markedly between seasons.

Understanding the conflict and perceptions of local communities about jackals is a key to improving the actual predation impact of jackals and to develop a sustainable management strategy. However, there is a paucity of information on the perceptions of local communities towards jackals, predation and associated management actions. Research has shown that many factors influence the conservation perceptions of local people positively or negatively [53]. In developing countries, education, gender, age, religion and ethnicity are important predictors of conservation perception, among demographic variables [54].

In the present study area, mixed farming, crop cultivation, and livestock rearing were the principal income sources for people living around the forest. The problem is more severe where most residents depend on mixed farming. Thus, respondents who derived income from farming and livestock tended to hold negative perception towards conservation of jackals. This might be related to the economic importance of livestock, which plays a vital role in the local people economy. Living alongside jackals might have caused a variety of additional costs aside from the direct impact of depredation, as people have to invest more heavily in strategies such as livestock herding, guarding and predator control. This might result in unfavorable perceptions toward jackals conservation among local people in the present study. This finding agrees with most of the studies (e.g., [13, 55, 56]. Within the assessment villages, jackals are hunted, trapped and illegally poisoned for their role as livestock predators. Persecution occurs in most villages as reported by the respondents as indicated by [57]. Therefore, decreasing livestock damage could be a conservation strategy.

Education is the most important factor influencing local people’s perceptions of the carnivore population in Ethiopia [58]. Findings of the present study also indicated that those respondents who were educated had a more positive perception towards the conservation of jackals than those with less or no education as reported in [55, 59]. As education level increases, negative perception towards conservation activities decreases. Therefore, awareness-raising education should be given for local people around KPCCF.

Age significantly influences the perception of the local community towards conservation. The young and middle aged class showed positive perception than the old age class. It might be due to the fact that younger respondents were more educated than the old age class and understand the value of wildlife in the area. The low awareness level shown by the older age group might have been caused by a lower education level, as indicated by [53, 60]. The older respondents might feel that the community forest would threaten their economy by reducing access to expanded farming and pasture land, settlement, fuel wood collection and extraction of forest products. Therefore, livestock damage, education status and age are the most influential determinants of the perceptions of local people toward jackals.

Human–jackal conflict might be related to increased jackal populations, proximity to human settlements and human activities like deforestation, fragmentation of forest, expansion of agricultural field and overgrazing in the study area. Human–jackal conflict could also be related to less availability of food resources in the forest and perhaps more dependency of jackals in the vicinity of village areas, ultimately provoking and aggravating the human-jackal conflict. [61] reported that increasing competition for natural resources is the leading cause of human-wildlife conflict in Kafta-Shiraro National Park, Northern Ethiopia. [53] also indicated that increasing the wildlife population from time to time and human proximity to protected areas are also the causes of human-wildlife conflict in and around Saint Borena National Park, Northern Ethiopia, and [62] conducted that increasing anthropogenic pressure in the area increases wildlife conflict in Abijata Shala Lake National Park, Ethiopia.

The conflict between wildlife and farmers significantly impacted rural people’s livelihoods [42]. The study revealed that jackals were considered the major notorious conflict animal by most respondents in the KPCCF. Our findings demonstrate that predation by jackal is a serious issue in all villages surveyed around KPCCF. Thus, the high consumption of domestic animals by jackals might be due to the low density of small and medium-sized wild animals in the study sites. [11, 40, 63], mentioned that jackals were the most frequently culpable for livestock losses among farmers living around the protected area. The results of the jackal scat sample analysis also agree with the respondents’ response. It showed that a large percentage of domestic animal species remain in scat of jackals.

The most frequently depredated domestic animals by jackals were cattle, sheep and poultry. Donkey, mule, and horse have also been reported in the diet of the black-backed jackal. This might be the cooperative hunting behavior of jackals; larger groups might help them subdue large-sized and difficult prey. Poor health conditions of these large-sized animals due to old age or sickness might also expose them to predators as reported by [3]. Jackals are, however, also adept at scavenging carrion and are known to feed on carcasses around cheetah (*Acinonyx jubatus*) and spotted hyenas [64, 65]. Given that this was a scat analysis, the researchers could not distinguish between carrion and fresh kills of these large-sized domestic animals. In addition, the researchers had observed jackals hunting sick cattle and attacking newborn calves. Different ecologists reported similar results in different parts of Ethiopia, where cattle, sheep, and poultry were the most frequently depredated animals [26, 32].

The scat analysis results also indicate that jackals have various food choices, from medium sized domestic animals to small prey. Similar studies were done in Ethiopia and Kenya by [2, 26]. Among domestic preys that analysed from jackal scats, sheep, cattle, poultry, and rodents were the diet of jackal followed by insects. The Ackerman index and Ivelves index conclude that sheep (+ 0.26), poultry (+ 0.05), and rodents (+ 0.33) were the most selectively preyed animals. The prevalence of domesticated animal and poultry remains in scats indicates the importance of these foods in jackals’ diets as well as their tendency to frequently visit human settlements in quest of food. Seasonal comparison indicated that domestic animals like sheep, cattle, and poultry, plant matter were more frequent in the wet season, and wild mammals like rodents and insects were frequent in dry seasons. Donkeys and horses were consumed by jackals, but to a much lesser degree than other animals. Similar results were conducted by [3, 48, 66]; the selection of these prey items as a food source for jackal might be primarily due to these prey items being readily available in the different study areas.

The domestic animal depredations by jackals were a commonly reported problem that responded from respondents in all study villages. The present study showed that jackals killed more domestic prey than wild ungulates. This might be a low abundance of alternative natural prey in the area or it might be ease of capturing domestic animals than wild preys [1, 3, 60]. A total of 624 (16%) kills of domestic animals were reported in the last five years. Livestock is an important source of protein, income, savings and social standing. Therefore, carnivore attacks on livestock are a major problem for rural communities. Our results indicate that livestock losses caused by jackals represent an economic concern for livestock owners in the KPCCF.In the last five years, the average economic loss due to jackal depredation was US$ 18,180.00, the average economic loss per year was US$ 3,636, and the annual mean economic loss per household was estimated to be US$ 12.5. [67] reported that the loss of domestic animals to jackal was 57.1% in and around the Simien Mountains National Park, Ethiopia; [40] reported 4.5% livestock kills by black-backed jackal and the economic loss was US $12,846 in Serengeti National Park, Tanzania. According to the study conducted by [10] in the Ambasil ecosystem of Kenya, the economic loss of livestock to black-backed jackal contributes 3.4% (the US $ 32,724.33). In Ethiopia, [55] reported that a total of 233 domestic animals (sheep, goat, cattle, donkey, and horse) were depredated in five years, the economic loss due to the depredations accounted for US$11,73.9 around Borena Sayint National Park, Ethiopia. The Focus Group Discussion revealed that livestock losses caused by jackal conflicts might represent an economic concern for livestock owners. This economic impact on an individual livestock owner might result in the retaliatory killing of the jackals in the KPCCF. Results from focus group discussions further revealed that the local people incur losses from jackals which not only kill their livestock but also jackals commonly interact with domestic dogs, and may serve as a vital link in the transmission of diseases like rabies and anthrax between wild animals and human settlement as reported by [67]. During Focus Group Discussion, the discussants also revealed that to mitigate human-jackal conflict various measures have to be taken by the forest management, such as providing awareness creation, initiatives on the community involvement in the forest management and compensation to the local community for the loss of livestock.

Most predation events occurred in the wet season (March to May 2020). This could be related to seasonal variations in wild prey dispersal. In addition to a suitable habitat cover for protection, the prey animals might secure their food nearby and limit their movement, minimizing exposure to predators during the wet season. As a result, predators may find it easier to prey on domestic animals in areas with low mean prey density. As a result, domestic animals in villages bordering the forest become an alternate food source. High livestock predation rates during the wet season due to low natural prey abundance have been reported in several studies by [26, 60]. During the dry season, food was scarce in the forest, and there might be a human disturbance and, domestic animal pressure in the forest and less agricultural activity near the forest, as indicated by [32]; anthropogenic factors including collecting firewood, habitat disturbance, agricultural expansion, and grazing land was a significant impact on the diet of jackals. Due to this impact, the wild herbivores might tend to concentrate near water sources in the protected area, and it is easier for jackals to prey on them. The diet composition confirmed that domestic animals were more frequent in wet seasons (45.5%) than wild mammals in the dry season (31%). In similar studies conducted by [3] in the Free State Province, South Africa, the domestic livestock (25–48%) were the most consumed ungulate, in winter (48%) and spring (46%), and wild ungulates (5%) consumed in spring and autumn in the diet of the jackal.

In many parts of Africa, the conflict between local people and wildlife is one of the most serious problems where villagers are located adjacent to nature reserves [4, 55]. The present study also shows that living in close proximity to protected areas imposes costs such as loss of livestock to jackal as noted by [4]. In villages close to the forest such as Wonchiro, Mehal and Damota high levels of predation were recorded and attacks occurred irrespective of season. The economic damage per household per year was also high in the village close to the forest boundary.

Human-wildlife conflict is a complex problem, requiring a combination of approaches to manage the conflict [4]. Our findings revealed that there is no single solution for mitigating livestock depredation around KPCCF but that a combination of predation management methods appears to be effective. The local community in the present study area deployed various traditional methods to protect their livestock from predators. They used repellents in the form of fire, noise, construction of different physical barriers, fencing, chasing, and guarding, a fear-provoking stimulus such as scarecrows are repellents for deterring predators as reported by [4, 31]. According to [59], guarding was likely to be more effective against carnivores. This was also true in the study area. Respondents indicated varying views regarding the effectiveness of wildlife damage control methods. When used with other methods, they perceived that guarding was quite effective method. Manual guarding as a most widely used protection measure was also reported by [61]. Without adequately addressing human–jackal conflict to conserve jackals and their habitat, conservation efforts will lose stability, progress and the support of local communities. Developing effective and well-targeted conservation strategies depends on fully understanding the complexities of the local situation in order to develop truly appropriate and practical solutions to conserve jackals. If farmers properly implement the numerous strategies used to limit predator activity around KPCCF, it could serve as a foundation for conflict mitigation. Our result could serve as a base line for KPCCF authorities, government agencies, and non-governmental organizations to create strategies to reduce conflict between people and jackals and to improve wildlife conservation in the KPCCF.

## Conclusions

Human- jackals conflict coupled with low-level awareness led to an increased negative human perception towards jackals with potentially adverse effects for conservation. Livestock depredation by jackals was the common conflict issue in the KPCCF. The results of this study indicate that jackal diets in the KPCCF have observed domestic animals as prey items. The diet composition in dry and wet seasons confirms the existence of livestock depredation in the area. The majority of respondents and Focus Group Discussion indicate that the increasing jackal populations in the study area, livestock losses, habitat fragmentation or losses, were the significant causes of human-jackal conflict. The estimated economic cost of domestic animals lost due to predation of jackal was US$18,180 in the last five years. This economic impact of the domestic animal depredation is high for livestock owner in the area, and which might provokes retaliatory killing of jackals. Local people close to the forest boundaries were highly vulnerable to the jackal. Most respondents in the study village responded that guarding was a more effective traditional method used by local farmers to reduce human-jackal conflict. The present study provides baseline information on some ecological aspects of jackals and their economic impact on livelihood around the KPCCF. As a considerable proportion of domestic stock was found in the diet of jackals in the current study, managing jackals in the KPCCF is a complex issue as they exert considerable ecological influence on ecosystems and also result in possible livestock losses for farmers. As human populations in the KPCCF keep encroaching on wildlife habitat, there will be more interaction between domestic animals and jackals. In addition to the ecological and financial impacts of their feeding activities, jackals commonly interact with wild carnivores, and this tendency may have significant epidemiological ramifications. They may thus serve as a vital link in the transmission of diseases like rabies between wild animals and human settlements. The present study could be used as a basis for developing a sustainable conservation strategy in the KPCCF that promotes human–jackal coexistence. Improved livestock husbandry methods, such as implementation of guarding animals, improving enclosures and herders and various other methods will be implemented by the local people for effective jackal conservation in the area. These findings also suggest further studies on the population size and feeding ecology of jackals and the resolution of human-jackal conflicts in the study area.

## Data Availability

The data used and analyzed during the current study are available from the corresponding author upon request.
